# Promoting Hepatitis B Awareness: Evaluating an Educational Approach through Health Care Workers in Tanzania

**DOI:** 10.5334/aogh.3045

**Published:** 2021-02-25

**Authors:** Nasreen S. Quadri, Shemal M. Shah, Holly Rodin, Jose D. Debes

**Affiliations:** 1 Department of Medicine, University of Minnesota, 420 Delaware Street SE, Minneapolis, Minnesota, USA; 2 Department of Medicine, Regions Hospital, 640 Jackson St, Saint Paul, Minnesota, USA; 3 Analytic Center of Excellence, Hennepin Healthcare, 701 Park Avenue, Minneapolis, Minnesota, USA; 4 Department of Gastroenterology and Hepatology, Hennepin Healthcare, 701 Park Avenue, Minneapolis, Minnesota, USA; 5 Arusha Lutheran Medical Center, Makao Mapya Road, Arusha, Tanzania

## Abstract

**Background:**

Hepatitis B virus (HBV) infection disproportionally affects populations in sub-Saharan Africa. Lack of HBV awareness perpetuates disease burden in Africa.

**Objective:**

To promote HBV awareness in Tanzania using a systematic, measurable, and expandable approach to educating health care workers (HCW).

**Methods:**

We designed and implemented an HBV knowledge and teaching skills session in southern Tanzania to empower HCWs in leading education to promote disease awareness in their communities. Training was divided into two sessions: didactic and practical. A five-question anonymous survey was distributed in person immediately before and after the practical portion of the training to evaluate HBV knowledge as well as specific skills for teaching. Differences between responses before and after the sessions were evaluated by Chi-Square analysis. A sub-group of questions were further analyzed for differences based on HCW self-report of HBV serostatus awareness.

**Findings:**

130 HCWs participated in the didactic lecture and 30 HCWs participated in both portions. A pre-post training five-question survey showed an increase in correct answers for all questions, with two showing statistical significance: HBV is silent (7% pre vs. 87% post; p < 0.0001), and repetition as key to promote awareness (63% pre vs. 100% post; p = 0.0002).

**Conclusions:**

Our low-cost intervention is applicable to increase HBV awareness in low resource settings across Africa.

## Background

Despite the introduction of universal vaccination and effective antiviral therapies, hepatitis B infection (HBV) still causes a high burden of disease in sub-Saharan Africa [1]. Unlike other chronic infectious diseases (i.e., HIV), there is a lack of awareness about HBV in communities across the continent. Indeed, data from our studies and others have found gaps in African health care workers’ (HCW) knowledge about HBV [2]. Awareness about HBV among the general public is even lower; globally, roughly 10.5% of people with chronic hepatitis B infection know their HBV status [3]. In chronic HBV, awareness is critical as the majority of infected individuals are asymptomatic until they develop cirrhosis or hepatocellular carcinoma (HCC), at which point those living in resource-limited settings are presented with very few options to prolong survival or improve quality of life. Community education is an essential, durable and sustainable solution to tackle the needs of preventing HBV and its complications; education can lead to vaccination and testing with appropriate linkage of patients to health care before irreversible sequelae of HBV occur [1]. However, promoting disease awareness in low resource settings is challenging. Barriers include low perception of personal risk, stigma, and priority of emergency over preventive health-seeking behavior [4]. Even among screened populations with self-reported awareness of HBV-positive status, 43.4% did not follow up with a health care professional for monitoring or treatment [5]. The implementation gap between health facility interventions and community access can be addressed by community facilitated participatory learning, as proven in the areas of maternal and newborn health, to ultimately improve health-care-seeking behavior and health outcomes if grounded in the local realities of the community [6]. We approached this challenge by designing a workshop to empower HCWs with high yield information about HBV while highlighting skills for teaching and engagement at the community level. Our approach relied on empowering HCWs at every level with specific HBV knowledge and teaching skills. HCWs are trusted members of society and can play an important role in increasing awareness in their respective communities.

## Methods

### Training session and setting

We designed and implemented a training session consisting of two portions: an initial didactic lecture on the current state of HBV globally and a practical “training the trainer” approach strategically scheduled after the didactic lecture. The session was implemented in Iringa, southern Tanzania, in the setting of an annual conference with 130 multi-disciplinary attendees from several regions of the country. The first portion described multiple aspects of HBV and was performed in a one-hour period. It focused, as described below, on expanding knowledge regarding the disease, intended for a professional audience of HCW with use of medically accepted language. The didactic lecture reviewed HBV epidemiology as well as the impact of its complications: cirrhosis and hepatocellular carcinoma, emphasizing that 25% of HBV-related deaths worldwide occur in Africa. We briefly covered diagnosis using specific testing modalities and indications for treatment and therapy options, particularly those available in the region. We emphasized both how to protect the patient living with HBV, promoting simple cares to minimize progression of disease, and how to protect close contacts. We counseled on specific interpersonal activities that do not transmit the virus and we countered misinformation about how the virus is spread amongst individuals. We further focused on preventable targets (screening for HCC, decrease in alcohol intake, etc.) sharing evidence-based tools that could be of use in resource-limited areas.

**Figure 1:**
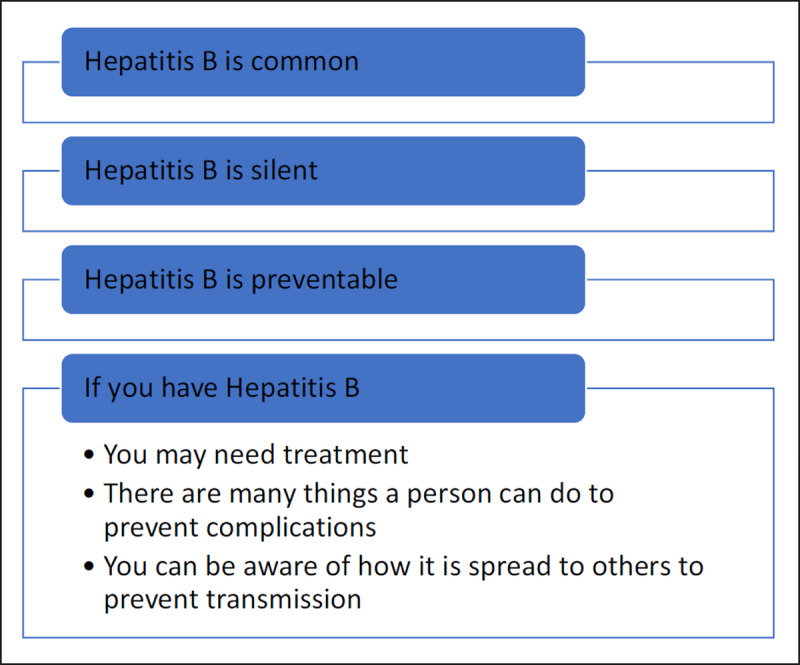
Workshop Messaging.

The second portion, dividing participants into small groups, focused on approaches to teaching about HBV using common language to engage the community. This portion was interactive and used adaptive teaching methods. The specific objective was to provide visual tools for HCWs to increase HBV awareness in their settings. Four major health messages about HBV were taught: hepatitis B is common, silent and preventable, and complications can be manageable. Further specifics emphasizing each point included: a) A visual of the HBV prevalence in Tanzania displaying 8% or 8 in 100 Tanzanians has HBV; b) description of how the virus typically does not cause symptoms until it has progressed late in the course with sequelae of cirrhosis or hepatocellular carcinoma; c) Prevention described in two-fold: knowledge about transmission and universal vaccinations for newborns, health care professionals and ideally the large majority of the population; d) Management of HBV, focusing on prevention of complications including avoidance of smoking, alcohol and aflatoxins. Skills shared about teaching included speaking clearly, intentionally using short sentences, emphasizing through repetition, engaging participants in active learning, and practicing teaching techniques frequently. An example short 3-minute video of a physician teaching about HBV was shown during the workshop, with a corresponding outline of talking points to emulate. Slides as visual-aid support were also provided. Participants were encouraged to disseminate information in clinic or dispensary waiting rooms where patients in sub-Saharan Africa already spend large periods of time awaiting medical consultation or assistance. The remainder of the workshop was utilized to address participant questions and distribution of health education handouts to be used in their individual health systems (***Figure 1***).

### Survey design and analysis

To evaluate immediate effectiveness of the teaching session, a 5-question multiple choice survey covering HBV knowledge and teaching techniques was administered before and after the workshop. Demographic information gathered on the survey included age, sex, profession and awareness of personal HBV serostatus. The survey consisted of five questions examining different aspects of HBV teaching and knowledge, including amplifying the message through repetition, importance of serostatus awareness, approach to teaching, common HBV complications in Africa, and vaccination. The surveys were distributed in person and filled out on paper immediately before and after the second portion of the teaching session, and were kept anonymous and collected in bulk to ensure the lack of visual identification of respondents. Differences between responses before and after the sessions were evaluated by Chi-Square analysis. A sub-group of questions were further analyzed for differences in comparison to HCW self-report of HBV serostatus awareness.

## Results

### Survey analysis

A total of 130 multidisciplinary HCWs were involved in the first portion of the teaching session and 30 HCWs were involved in both the didactic and practical portions. The latter cohort completed the written survey and consisted of 25 nurses, 3 doctors and 2 pharmacists. As expected, all respondents performed better on the post-workshop test (***Table 1***). In two of the survey questions, the improvement was statistically significant. This included evaluating that HBV is silent (7% pre-course vs. 87% post-course; p < 0.0001), and repetition as key to promote awareness (63% pre-course vs. 100% post-course; p = 0.0002). Of the participants,100% of doctors, 60% of nurses and 50% of pharmacists self-reported awareness of personal HBV serostatus. Interestingly, after correction for self-reported awareness of HBV status, the differences remained significant for both of those questions (11% pre-course vs. 86% post-course, p < 0.0001) and (63% pre-course vs. 100% post-course, p = 0.009), respectively. Four health care workers corresponded with questions and updates about educational sessions in their setting after an email was sent to all workshop participants one month later.

**Table 1:** Survey results evaluating hepatitis B knowledge and teaching skills.


	PRE-WORKSHOP CORRECT (N/TOTAL, %)	POST-WORKSHOP CORRECT (N, %)	P VALUE

**Question #1^a^**	2/29, 7%	26/30, 87%	p < 0.001

**Question #2^a^**	25/30, 83%	27/30, 90%	NS^b^

**Question #3^a^**	19/30, 63%	30/30, 100%	p = 0.0002

**Question #4^a^**	17/30, 57%	24/30, 80%	NS^b^

**Question #5^a^**	23/30, 77%	27/30, 90%	NS^b^


^a^ 1) Which one of the following messages about hepatitis B is critical to tell the patient when counseling? (HBV is silent). 2) The importance of a person knowing about their hepatitis B status (prevent complications). 3) A simple and useful approach to teach about hepatitis B (repetition). 4) The most feared complication of hepatitis B in Africa (liver cancer). 5) Which of the following is NOT true (there is no vaccine for HBV). ^b^ NS—Not Significant.

## Discussion

Our approach provides a low-cost HCW-led model that allows scalability and sustainability to promote HBV awareness, ensuring that knowledge and tools for dissemination can reach a wide array of communities in sub-Saharan Africa. HCWs are familiar with their communities from their intimate work alongside patients and families in their individual settings and have gained the respect of their communities. The trust, language compatibility and familiarity with the landscape all make local HCWs the ideal leaders for sharing educational materials. Moreover, they also have a familiarity of medical facilities in the community for specialty referral, knowledge of supply for vaccinations in the region, and appropriate laboratories for testing referrals. However, challenges do remain: most notably, critical assessment of unequal conditions that foster poor health, with resources to address the structural determinants; the perceived and real power dynamics between HCWs and the communities they serve; and financial support to continue community level programming [6]. We believe that resources centered on education will enable adoption of all other strategies for hepatitis B elimination such as universal vaccination, HBV screening, linkage to care for diagnosis and treatment, prevention of mother to child transmission and prevention of adult acquisition, similar to other infectious diseases, like HIV [7]. This low-cost, one-time intervention methodology is easily applicable to other regions of the continent, particularly in low resource settings, with the ultimate goal of tackling morbidity and mortality from HBV in sub-Saharan Africa.

## Supplementary File

The Supplementary File for this article can be found as follows:

10.5334/aogh.3045.s1 Example Instructional 3-Minute Video Teaching about Hepatitis B (HBV). This video file is the example instructional video shared during the second portion of the workshop, which offered participants an example of how a physician would teach about Hepatitis B in the community with attention to talking points to emulate. The video file was shared with participants at the conference to revisit upon return to their institutions.

## Appendix

### Hepatitis B Workshop Survey

(please circle the best answer)

-What is your age? ____

-Are you: Female ____ or Male ____

-Are you:

              Doctor ___ Nurse ___ Pharmacist__Administrator__ Other___

-Do you know your hepatitis B status: Yes ___ No ___

1. Which one of the following messages about Hepatitis B is critical to tell the patient when counseling?
  - Hepatitis B is important
  - Hepatitis B can kill
  - Hepatitis B is silent
  - Hepatitis B affects the liver
2. The importance of a person knowing about their Hepatitis B status is that:
  - They can improve their diet
  - They can take steps to prevent transmission to others and complications
  - They can take medications for hepatitis B
  - They can inform work of their status
3. A simple and useful approach to teach about Hepatitis B is:
  - To repeat the message
  - To use medical language
  - To speak loudly
  - To sound confident
4. The most feared complication of Hepatitis B in Africa is:
  - Liver Cirrhosis
  - Gastrointestinal bleed
  - Liver cancer
  - Ascites
5. Which of the following is NOT true:
  - Hepatitis B is very common in Tanzania
  - There is no vaccine for hepatitis B
  - Hepatitis B can lead to liver cancer
  - Patients with Hepatitis B should not drink alcohol
